# Nectin-4 expression in vulvar squamous cell carcinoma

**DOI:** 10.1007/s00428-026-04495-1

**Published:** 2026-03-26

**Authors:** Frederik A. Stuebs, Antje Knöll, Arndt Hartmann, Christian Matek, Nelson John, Lothar Häberle, Matthias W. Beckmann, Carlos Bercebal, Markus Eckstein, Sebastian Rauch, Carol I. Geppert

**Affiliations:** 1 Department of Gynecology and Obstetrics, Bavarian Cancer Research Center (BZKF), Erlangen University Hospital, Comprehensive Cancer Center, Friedrich-Alexander-Universität Erlangen-Nürnberg, Universitaetsstrasse 21–23, Erlangen, 91054 Germany; 2 Virologisches Institut – Harald-zur-Hausen-Institut für Virologie, Uniklinikum Erlangen, Friedrich-Alexander-Universität Erlangen-Nürnberg, Schlossgarten 4, Erlangen, 91054 Germany; 3 Institute of Pathology, Erlangen University Hospital, Comprehensive Cancer Center Erlangen–European Metropolitan Area of Nuremberg (CCC ER-EMN), Bavarian Cancer Research Center (BZKF), Erlangen, Friedrich-Alexander-Universität Erlangen-Nürnberg, Krankenhausstrasse 8–10, Erlangen, 91054 Germany; 4 Biostatistics Unit, Department of Gynecology and Obstetrics, Comprehensive Cancer Center Erlangen-EMN, Friedrich-Alexander University Erlangen-Nuremberg, Erlangen, Germany

**Keywords:** Vulvar squamous cell carcinoma, Nectin-4, Antibody-drug conjugates, Immunohistochemistry, Fluorescence in situ hybridization

## Abstract

Therapy options for women with vulvar squamous cell carcinoma (VSCC) are limited. New strategies such as immune checkpoint inhibitors (ICI) or antibody-drug conjugates (ADC) are lacking. One potential target for the ADC Enfortumab Vedotin (EV) is Nectin-4. EV has been successfully used to treat metastatic urothelial carcinoma (mUC). Currently, there is limited data on the expression of Nectin-4 for VSCC. Our aim was therefore to investigate the expression of Nectin-4 in women with VSCC. In total, 219 formalin-fixed paraffin-embedded tissue (FFPE) samples of primary VSCC were collected between 2000 and 2021. A next generation tissue microarray (ngTMA) was constructed. The assessment of Nectin-4 expression was performed through the implementation of immunohistochemistry (IHC). We also performed NECTIN-4 fluorescence in situ hybridization (FISH) to evaluate amplification of Nectin-4. The median Nectin-4 H-score for membranous expression was 22.5, with 32.4% of VSCCs exhibiting moderate to strong expression. We could not show that Nectin-4 H-score (membranous) significantly influenced overall survival in addition to other prognostic factors. The rates of regional lymph node metastases, lymphatic vascular space involvement, G3-Grading, perineural sheath invasion and p16-/p53+ were highest in Nectin-4-negative VSCC. 28.8% of recurrent VSCC had moderate or strong expression of Nectin-4. None of the investigated VSCC (primary or recurrent) showed amplification of Nectin-4 in contrast to mUC. Nectin-4 could be a potential target in the therapy of women with VSCC. Further (pre-)clinical data is needed to evaluate the use of EV in therapy of women with VSCC.

**Supplementary Information:**

The online version contains supplementary material available at https://doi.org/10.1007/s00428-026-04495-1.

## Introduction

Vulvar cancer (VC) accounts for 4–5% of gynecologic cancers and 0.3% of all newly diagnosed neoplasms and is a rare malignancy of the lower genital tract [1, 2]. In 2022, 3144 women were newly diagnosed with VC in Germany [3]. The majority of women diagnosed with VC are over the age of 70. The incidence of VC has increased progressively in Germany over the last decades. The greatest increase in incidence has been reported in females under the age of 70 [4]. The most common histologic type of vulvar cancer is vulvar squamous cell carcinoma (VSCC) [1]. VSCC is caused by two different pathologic mechanisms [5]. The most common type of VSCC is associated with chronic dermatoses such as lichen, which are common in elderly women (HPV-independent (HPV-)). The second oncogenic pathway is associated with persistent infection with high-risk human papillomavirus (HPV) (HPV+). Women diagnosed with HPV+ VSCC tend to be younger at diagnosis [6]. The 2-year overall survival (OS) for HPV+ VSCC has been reported to be up to 82.5% compared to 70.4% for HPV- VSCC [7].

Treatment options for women with VSCC in early-stage disease are limited to surgical resection of the tumor. Depending on the stage of the tumor, adjuvant radiotherapy or radio-chemotherapy is performed [8–10]. When the disease is advanced, resection is often not possible, so primary radio-chemotherapy must be administered. In rare situations, exenteration can be performed. The prognosis of the patients decreases significantly with increasing tumor stage. In the metastatic setting, mono-chemotherapy is often the only remaining treatment option [9]. There is an urgent need for new therapeutic strategies to improve outcomes for women with VSCC. Although immune checkpoint inhibitors (ICIs) and antibody-drug conjugates (ADCs) have transformed the treatment landscape for many malignancies and opened up new therapeutic options, data on VSCC are lacking [11–14].

A possible target for ADC is Nectin-4 [15]. Nectin-4 is a part of the Nectin-family of adhesion molecules. They mediate Ca^2+^-independent cell–cell adhesion [15]. Enfortumab Vedotin (EV) is targeting Nectin-4 and is approved for the treatment of metastatic urothelial cancer (mUC) [16]. Pretreatment screening for expression of Nectin-4 is not mandatory, but recent data suggest a correlation between expression levels of Nectin-4 and treatment response to EV. Absence or weak expression of Nectin-4 in mUC was associated with a poor progression-free survival (PFS) [17]. In mUC with strong membranous Nectin-4 expression, amplification of Nectin-4 is frequently detected [18]. In penile cancer, a squamous cell carcinoma with HPV+ and HPV- carcinogenesis, 28.0% of primary tumors show either an intermediate or high expression of Nectin-4 [19].

So far, limited data exist on the expression of Nectin-4 in vulvar cancer [20]. VSCC is further not represented in TCGA at all. The current study aims to investigate the expression of Nectin-4 in cytoplasm and membranous as well as the rate of amplification in one of the world’s largest VSCC collectives. These rates of expression were correlated with clinicopathologic characteristics such as tumor stage, grading, tumor infiltrating lymphocytes (TIL), and overall survival.

## Material and methods

### Allocation of biomaterial

In total 219 formalin-fixed paraffin-embedded tissue (FFPE) samples of primary VSCC and 59 samples of recurrent VSCC were collected between 2000 and 2021. Clinical data such as TNM-Stage, tumor grade, depth of invasion, venous invasion, lymphatic invasion and perineural sheath invasion were assessed in a standardized manner using routine pathology and clinical data reports. Subtypes, tumor growth and any immunohistological markers were described as reported previously [21, 22].

### Preparation of tumor material of VSCC

The construction of next generation tissue microarray (ngTMA) has been described in detail previously [23]. In brief, the FFPE samples were scanned under a microscope and slides were selected that represented individual tumor tissue best for each case. Those were used for the ngTMA. The ngTMA encompasses the domain of digital pathology and the utilization of annotation-guided automated arraying techniques for organizing all samples in alignment with the prescribed tissue processing steps [21, 22].

### Immunohistochemistry

**Fig. 1:**
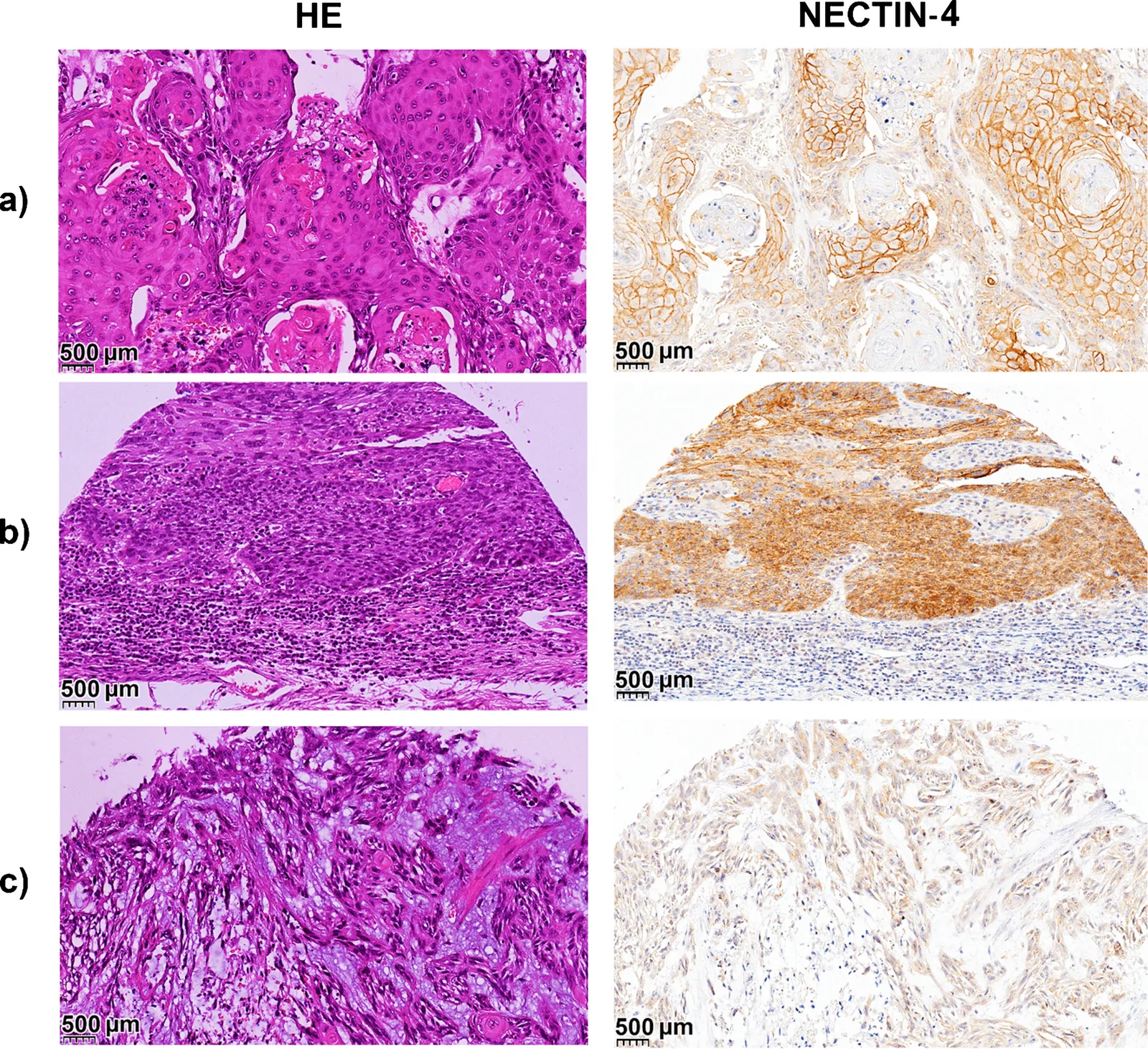
**a** Well differentiated keratinizing squamous cell carcinoma, in which the NECTIN-4 staining shows both a high intensity membrane positivity and a low intensity cytoplasmatic positivity. **b** In this case the predominant staining pattern is cytoplasmatic (moderate to high intensity), even though a low intensity membrane positivity can also be identified. **c** Poorly differentiated (G3) non-keratinizing squamous cell carcinoma composed of discohesive neoplastic cells surrounded by a fibro-myxoid stroma. An example of how poorly differentiated tumours may lose NECTIN-4 expression. Notwithstanding, low intensity cytoplasmatic positivity is still retained in some cells. Pictures taken at 200× magnification, 0.221 µm/pixel; scale bar: 500 µm; ndpi format; scanned by NanoZoomer S210 from Hamamatsu (C13239 MI), NDP.Scan vers. 3.4.1 and demonstrated by NDP.view 2, Hamamatsu, Hamamatsu-City, Japan. For magnified examples of keratinizing and non-keratinizing VSCC see supplement Figure S1a-c

The assessment of Nectin-4 expression is performed through the implementation of immunohistochemistry (IHC). This approach is a well-established method in the field of pathology, particularly in the context of mUC diagnostics, as it is routinely employed in the department of pathology at Erlangen, accredited and certified according to DIN EN ISO/IEC 17020 [17]. Staining procedures for Nectin-4 were performed on a VENTANA BenchMark Ultra autostainer according to vendors standards and protocols. Membranous NECTIN-4 expression was evaluated by experienced (board-certified) senior gynecologic pathologist (C. Geppert/A. Hartmann). The localization of NECTIN-4 immunoreactivity was determined in tumor cells, with specific expression in cell membrane and cytoplasm. However, the study focused on the assessment of specific membranous expression as the biological prerequisite for EV binding. The samples were then classified as “negative” (H-score 0–14), “weak” (H-score 15–99), “moderate” (H-score 100–199), or “strong” (H-score 200–300) (see Fig. 1) [17]. The immunochemistry for tumor infiltrating lymphocytes (TIL) has been described before [21, 22]. The important molecular subtypes for VSCC were characterized for HPV-dependent (p16+/p53 wildtype), HPV-independent (p16-/p53 mutated) individuals and as well as in a third group of p16-/p53- VSCC as previously described [21, 22].

#### NECTIN-4 fluorescence in situ hybridization

**Fig. 2:**
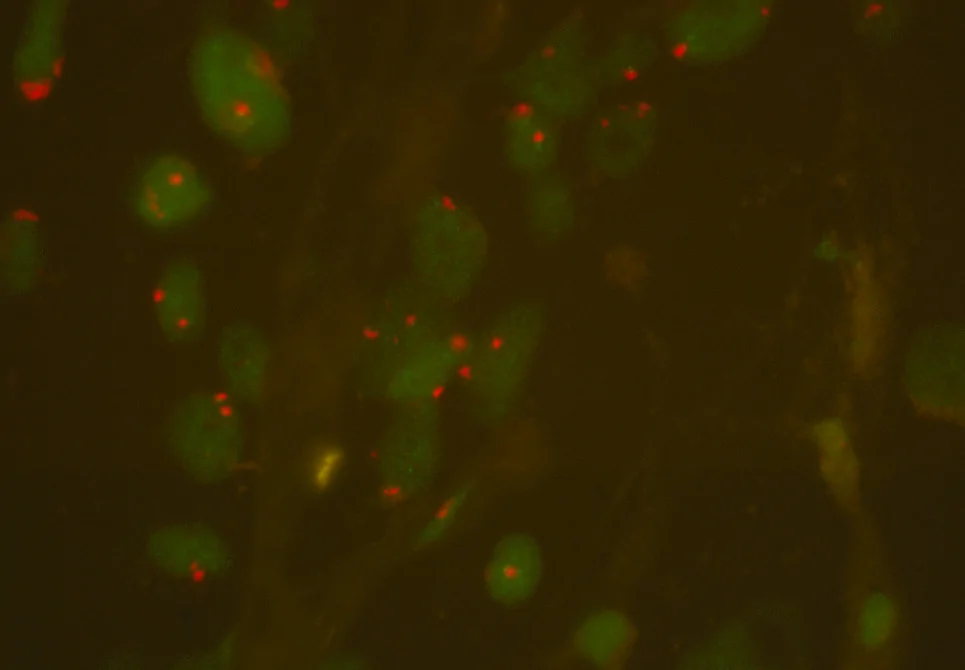
Nectin-4 FISH staining in a keratinizing squamous cell carcinoma with no amplification for Nectin-4 in the tumor cells. TMA 20–21, core 10; centromere signals in red, Nectin-4 signals in green. Pictures taken at 630× magnification (oil, raw:2088 × 1450 pixel; scale bar: 50 µm; camera: Gryphax Arktur Jenoptik, Jena, Germany, microscope: Leica DM 4000B, Leica, Wetzlar, Germany)

The NECTIN-4 fluorescence in situ hybridization (FISH) was performed in 140 cases using a probe from Empire Genomics (Catalog No. NECTIN-4–20-GR, Empire Genomics, Buffalo, NY) [18]. The probe is designed to specifically target and bind to the NECTIN-4 gene (NCBI Gene ID: 81607). The probe consists of a fluorescently labeled DNA probe that has been engineered to bind specifically to the NECTIN-4 gene [18]. Representative tumor areas for formal analysis were selected by an experienced board-certified pathologist (CG; blinded to patient outcomes), and at least 50 nonoverlapping nuclei per sample were assessed. The intensity of the green (NECTIN-4) and red (CEN1) signals was subsequently quantified manually. The NECTIN-4/CEN1 ratio was subsequently calculated, and a ratio of ≥2.0 was deemed indicative of NECTIN-4 amplification. Conversely, tumors exhibiting a ratio value less than 2.0 were designated as nonamplified (see Fig. 2) [18].

## Statistical analysis

Statistical analysis was done for primary VSCC only. Patient and tumor characteristics are described using appropriate summary statistics. The mean and standard deviation as well as the median and the interquartile range (25th and 75th percentiles) are calculated for continuous variables, while frequency and percentage are used for categorical variables.

Differences in membranous and cytoplasmic Nectin-4-H score across p16/p53 subtypes were assessed using Kruskal–Wallis tests. Median and interquartile ranges were calculated.

Overall survival (OS) time was defined as the period from the date of primary diagnosis to the date of death or the last date the patient was known to be alive. Recurrence-free survival (RFS) time was defined in a similar fashion. For both endpoints, follow-up was administratively censored at 10 years.

The impact of Nectin-4–H score membranous and cytoplasmic on OS in addition to the established prognostic factors (age, depth of invasion, primary tumor stage, regional lymph nodes, grading, p16/p53 subtype) was tested using likelihood ratio tests that each compared two multivariable Cox regression models, one model with the established prognostic factors and another one with those prognostic factors and additionally Nectin-4–H score membranous and Nectin-4-H score cytoplasmic, respectively.

The adjusted hazard ratios (HR) for Nectin-4–H score membranous and cytoplasmic expression were estimated. As sensitivity analysis, corresponding unadjusted HRs were estimated. OS and RFS rates with 95% confidence intervals (CIs) were estimated using the Kaplan–Meier product limit method.

The proportional hazards assumptions were checked using the Grambsch-Therneau method. Missing values for other predictors were imputed as done in Salmen et al. [24].

All of the statistical tests were two-sided, and a *P* value < 0.05 was regarded as statistically significant. To address the problem of multiple testing, the P values of the statistical tests described above (four tests) were corrected using the Bonferroni method. Both raw and Bonferroni corrected *P* values were reported. *P* values were calculated only for primary study aims to avoid false-positive results.

Calculations were carried out using the R system for statistical computing (version 4.3.0; R Development Core Team, Vienna, Austria, 2023).

## Results

### Patient characteristics

A total of 219 patients with primary VSCC were analyzed. Patient characteristics for patients with primary VSCC are presented in Table 1 and the mean age was 64.4 years. The majority of women had a pT1b VSCC. Regional lymph node metastases were present in 31.6% of women, and 12.3% had lymphatic invasion. Among the VSCC cases, 52.8% were p16-/p53+, and 25.9% were p16+/p53-.

**Table 1:** Patient and tumour characteristics for patients with primary VSCC, showing mean and standard deviation (SD), median and interquartile range (IQR) or frequency and percentage

		All Patients (*N* = 219)
Age (Years)	Mean (SD)	64.4 (16.1)
	Median (IQR)	67.3 (51.4, 76.9)
Depth of invasion in cm	Mean (SD)	0.6 (1)
	Median (IQR)	0.4 (0.1, 0.7)
Primary tumor stage	T1a	47 (21.5)
	T1b	150 (68.5)
	T2	14 (6.4)
	T3	7 (3.2)
	T4	1 (0.5)
Regional lymph nodes	N0	130 (68.4)
	N +	60 (31.6)
Venour invasion	V0	217 (99.1)
	V1	2 (0.9)
Lymphatic invasion	L0	192 (87.7)
	L1	27 (12.3)
Perineural sheath invasion	Pn0	192 (87.7)
	Pn1	27 (12.3)
Grading	G1	29 (13.3)
	G2	92 (42.2)
	G3	97 (44.5)
P16/P53 Subtype	p16-/p53-	46 (21.3)
	p16-/p53 +	114 (52.8)
	p16 +/p53-	56 (25.9)
PD-L1	< 1	69 (31.5)
	1 – < 10	59 (26.9)
	≥ 10	91 (41.6)

### Nectin-4-expression

The median Nectin-4 H-score (membranous) was 22.5 (IQR: 0.0 to 180.0) for p16-/p53-, 12.5 (IQR: 0.0, 103.8) for p16-/p53+ and 55.0 (IQR: 5.0 to 123.8) for p16+/p53-. The differences among the P16/P53 subtypes were not statistically significant (raw *P* = 0.14, Bonferroni corrected *P* = 0.56; Kruskal–Wallis test). The rates of regional lymph node metastases, lymphatic invasion, G3-Grading, perineural sheath invasion and p16-/p53+ were highest in Nectin-4-negative VSCC (see Table 2).

**Table 2:** Patient and tumor characteristics relative to Nectin 4 – H Score membranous (primary VSCC)

		NECTIN 4- H Score (membranous)			
		Negative (0–14) (***N*** = 98)	Weak (15–99) (***N*** = 50)	Moderate (100–199) (***N***= 43)	Strong (200–300) (***N*** = 28)
Age (years)	Mean (SD)	68.1 (15.2)	63.7 (14.8)	59 (16.4)	61 (18.2)
	Median (IQR)	69.7 (56, 80.2)	64.6 (51.8, 75.8)	61.1 (56, 80.2)	65 (51.8, 75.8)
Depth of invasion in cm	Mean (SD)	0.8 (1.3)	0.5 (0.6)	0.3 (0.2)	0.3 (0.3)
	Median (IQR)	0.5 (0.3, 1)	0.2 (0.1, 0.5)	0.3 (0.3, 1)	0.2 (0.1, 0.5)
Primary tumor stage	T1a	7 (7.1)	16 (32)	14 (32.6)	10 (35.7)
	T1b	78 (79.6)	29 (58)	26 (60.5)	17 (60.7)
	T2	9 (9.2)	3 (6)	2 (4.7)	0 (0)
	T3	4 (4.1)	2 (4)	0 (0)	1 (3.6)
	T4	0 (0)	0 (0)	1 (2.3)	0 (0)
Regional lymph nodes	N0	54 (59.3)	32 (84.2)	28 (73.7)	16 (69.6)
	N +	37 (40.7)	6 (15.8)	10 (26.3)	7 (30.4)
Venour invasion	V0	97 (99)	50 (100)	43 (100)	27 (96.4)
	V1	1 (1)	0 (0)	0 (0)	1 (3.6)
Lymphatic invasion	L0	82 (83.7)	46 (92)	38 (88.4)	26 (92.9)
	L1	16 (16.3)	4 (8)	5 (11.6)	2 (7.1)
Perineural sheath invasion	Pn0	79 (80.6)	46 (92)	39 (90.7)	28 (100)
	Pn1	19 (19.4)	4 (8)	4 (9.3)	0 (0)
Grading	G1	7 (7.1)	7 (14)	10 (23.8)	5 (17.9)
	G2	37 (37.8)	28 (56)	19 (45.2)	8 (28.6)
	G3	54 (55.1)	15 (30)	13 (31)	15 (53.6)
P16/P53 Subtype	p16-/p53-	20 (20.6)	7 (14.3)	10 (23.8)	9 (32.1)
	p16-/p53+	58 (59.8)	25 (51)	19 (45.2)	12 (42.9)
	p16+/p53-	19 (19.6)	17 (34.7)	13 (31)	7 (25)
PD-L1	< 1	30 (30.6)	14 (28)	15 (34.9)	10 (35.7)
	1 – <10	24 (24.5)	15 (30)	15 (34.9)	5 (17.9)
	≥ 10	44 (44.9)	21 (42)	13 (30.2)	13 (46.4)

The median Nectin-4 H-score (cytoplasmatic) was 15.0 (IQR: 0.0 to 38.8) for p16-/p53-, 20.0 (IQR: 0.5 to 70.0) for p16-/p53+ and 25.0 (IQR: 5.0 to 70.0) for p16+/p53-. Again, the differences among the P16/P53 subtypes were not statistically significant (raw *P* = 0.31, Bonferroni corrected *P* = 1.00; Kruskal–Wallis test). There was no clear pattern for the expression of cytoplasmatic Nectin-4 and histopathological features (see Table S1).

### Correlation with survival rates

We could not show that Nectin-4 H-score (membranous) significantly influences overall survival in addition to other prognostic factors (raw *P* = 0.51, Bonferroni corrected *P* = 1.00, likelihood ratio test). The adjusted HRs for overall survival were 0.80 (95% CI: 0.43–1.50) for the “weak” group versus the “negative” reference group, 1.18 (95% CI: 0.60–2.30) for the “moderate” group versus “negative” and 0.55 (95% CI: 0.23–1.32) for the “strong” group versus “negative”. Similarly, Nectin-4 H-score (cytoplasmatic) was not found to significantly influence overall survival in addition to other prognostic factors (raw *P* = 0.65, Bonferroni corrected *P* = 1.00, likelihood ratio test). The adjusted HRs for overall survival were 1.15 (95% CI: 0.71–1.87) for the “weak” group versus the “negative” and 1.40 (95% CI: 0.62–3.14) for the “moderate or strong” group versus “negative” (Table 4).

**Fig. 3:**
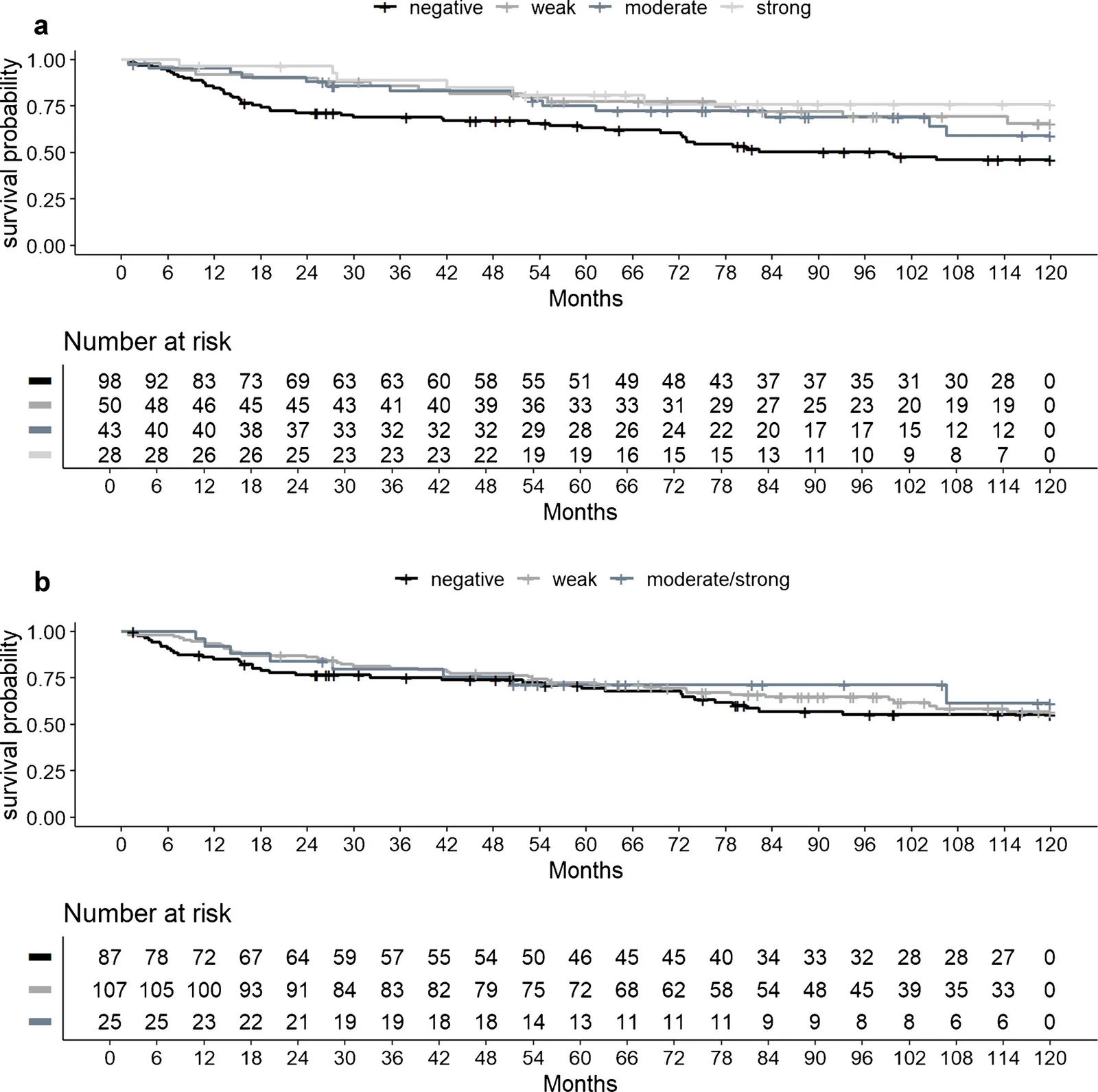
**a** Overall survival rates relative to NECTIN 4–H score (membranous). **b** Overall survival rates relative to NECTIN 4–H score (cytoplasmatic)

Unadjusted HRs and Kaplan–Meier survival rates are shown in Table 3, Table 4, and Fig. 3a and b.

**Table 3:** Overall survival rates and confidence interval (primary VSCC)

NECTIN 4-H Score		N	Events	2-year survival rate (95% CI)	5-year survival rate (95% CI)	10-year survival rate (95% CI)
Membranous	Negative (0–14)	98	48	0.71 (0.63–0.81)	0.63 (0.54–0.74)	0.46 (0.36–0.58)
	Weak (15–99)	50	15	0.90 (0.82–0.99)	0.77 (0.66–0.90)	0.66 (0.52–0.82)
	Moderate (100–199)	43	14	0.88 (0.79–0.98)	0.75 (0.63–0.90)	0.59 (0.44–0.80)
	Strong (200–300)	28	6	0.96 (0.90–1.00)	0.81 (0.67–0.97)	0.76 (0.61–0.95)
Cytoplasmatic	Negative (0–14)	87	34	0.77 (0.68–0.86)	0.69 (0.60–0.80)	0.55 (0.45–0.68)
	Weak (15–99)	107	41	0.86 (0.80–0.93)	0.72 (0.64–0.81)	0.57 (0.47–0.68)
	Moderate (100–199) or strong (200–300)	25	8	0.84 (0.71–1.00)	0.71 (0.56–0.92)	0.61 (0.41–0.91)

**Table 4:** Cox regression analyses for overall survival, showing adjusted and unadjusted hazard ratios for Nectin 4–H Score. (primary VSCC)

Nectin 4–H Score		Adjusted^1^ HR (95% CI)	Unadjusted HR (95% CI)
Membranous	Negative (0–14)	Reference	Reference
	Weak (15–99)	0.80 (0.43–1.50)	0.51 (0.29–0.91)
	Moderate (100–199)	1.18 (0.60–2.30)	0.59 (0.32–1.07)
	Strong (200–300)	0.55 (0.23–1.32)	0.38 (0.16–0.88)
Cytoplasmatic	Negative (0–14)	Reference	Reference
	Weak (15–99)	1.15 (0.71–1.87)	0.86 (0.54–1.35)
	Moderate (100–199) or strong (200–300)	1.40 (0.62–3.14)	0.78 (0.36–1.67)

*HR* hazard ratio, *CI* confidence interval

HRs were adjusted for age, depth of invasion, primary tumor stage, regional lymph nodes, grading, and p16/p53 subtype

#### Correlation with recurrence-free survival rates (RFS)

For 39 women primary and recurrent VSCC was available. Fifteen cases of recurrent VSCC showed no expression of Nectin-4, whereas 14 positive cases showed at least weak expression of Nectin-4. The vast majority of recurrences were local. Nectin-4 expression did not influence RFS in women with primary VSCC. In VSCC with Nectin-4 expression, the RFS was slightly better than in Nectin-4 negative VSCC (see Table 5 and Figure S2a and S2b).

**Table 5:** Recurrence-free survival rates and confidence interval

NECTIN 4-H Score		***N***	Events	2-year survival rate (95% CI)	5-year survival rate (95% CI)	10-year survival rate (95% CI)
Membraneous	Negative (0–14)	98	15	0.87 (0.80, 0.94)	0.84 (0.76, 0.92)	0.82 (0.74, 0.91)
	Positive (≥15)	121	14	0.92 (0.88, 0.97)	0.87 (0.81, 0.94)	0.87 (0.81, 0.94)
Cytoplasmatic	Negative (0–14)	87	12	0.92 (0.87, 0.98)	0.84 (0.76, 0.93)	0.82 (0.73, 0.92)
	Positive (≥15)	132	17	0.88 (0.83, 0.94)	0.86 (0.81, 0.93)	0.86 (0.81, 0.93)

#### Results of the FISH analysis

FISH was performed on all cases of VSCC with high Nectin-4 membranous expression. However, none of the Nectin-4 FISH results were positive, meaning that no Nectin-4 amplification could be detected.

No clear pattern in the correlation for Nectin-4 expression and TIL could be shown, neither for membranous nor cytoplasmic Nectin-4 (see Tables S1 and S2).

#### Comparison between primary and recurrent VSCC

Additionally, we also analyzed 59 cases of recurrent VSCC (some without primary VSCC available for comparison). For recurrent VSCC, 52.5% exhibited negative membranous Nectin-4 expression. The expression of Nectin-4 was found to be moderate to strong in 28.8% of cases. Cytoplasmic expression was observed to be moderate or positive in 20.3% of cases (see Table S2).

## Discussion

To the current date, new therapeutic strategies for women with VSCC such as ICI or ADC are lacking. In this particular context, ADC therapies, such as EV, have the potential to enhance the effectiveness of VSCC therapeutic interventions, thus overcoming the current therapeutic limitations for women with a poor prognosis. Therefore, the expression of Nectin-4 in women with VSCC was investigated in order to evaluate the presence of possible targets. In this study, we investigated 219 cases of primary VSCC by immunohistochemistry (expression level) and molecular pathology (amplification level). This is the first study to evaluate the expression of Nectin-4 in women with VSCC in a large study cohort with immunohistochemistry and FISH; 44.7% of VSCC had no membranous staining of Nectin-4 whereas 32.4% had a moderate or high expression of Nectin-4. Limited data exist for Nectin-4 expression in VSCC [20, 25]. In a series of 55 cases of VSCC all showed at least a weak Nectin4 expression. Moderate/high expression of Nectin-4 expression was more frequent in p16+/p53- VSCC (85.7%) when compared to p16-/p53+ VSCC (62.9%) [20]. In our study, moderate/high expression was also higher in p16+/p53- VSCC compared to p16-/p53+ VSCC 35% vs. 27.2%, respectively. In contrast to the data by Höhn et al. we could show the highest rates of moderate/high expression for p16-/p53- VSCC. Like Höhn et al. we analyzed Nectin-4 expression by IHC. The main difference from those experiments is the use of the H-score to evaluate Nectin-4 expression, which was done in a comparable way for penile cancer (PC) and urothelial cancer (UC), as mentioned above. Therefore, the results of our study can be compared more precisely with those of other tumor types. The H-score calculation method is widely used to establish links between proteins and tumors. It plays a crucial role in diagnosis, prognosis and therapeutic decision-making [26]. The expression of nectin-4 in penile cancer (PC) has been previously described. This tumor has a pathogenesis similar to that of VSCC, as it is also a squamous cell carcinoma that can be HPV-dependent or -independent [19, 27, 28]. Moderate or strong expression of Nectin-4 was found 23.4%–28% of PC [19]. Another study investigating Nectin-4 expression observed even higher levels of moderate and strong expression, reaching 79% [27]. The median H-score of membranous Nectin-4 expression was 30 at the invasion front in PC and therefore slightly higher compared to VSCC [19]. In a different study the median H-score was 140 for PC [27]. For VSCC membranous samples showed higher H-scores than cytoplasmic samples, which is different from the results seen in PC [19]. The observed variation in H-score may be attributable to the heterogeneous immunoreactivity present across different areas of the tumor, a finding that has been described before. This observation might have significant implications for the use of a ngTMA in evaluating Nectin-4 expression [27].

For PC with high expression of Nectin-4, a better prognosis was observed compared to low and intermediate expression [27]. In two different studies, no significant prognostic value of Nectin-4 in patients with PC was found [19, 29]. In our study, we observed better survival outcomes for women with high Nectin-4 levels, though these results were not statistically significant (*P* = 0.65). For PC, membranous Nectin-4 expression decreased significantly with an increase in the T-stage [19]. For VSCC, no correlation between expression of Nectin-4 and T-stage was observed. Regional lymph node involvement was more prevalent in Nectin-4-negative VSCCs than in those cases with moderate or strong Nectin-4 expression. For PC, no statistically significant difference in Nectin-4 expression was found regarding lymph node involvement [19]. In VSCC, higher rates of membranous Nectin-4 expression were observed in p16+/p53- compared to p16-/p53+ subtypes. Higher expression of Nectin-4 was also found in HPV-positive penile carcinomas [19, 27]. The observed discrepancies in expression outcomes among individual studies may be attributable to the heterogeneous nature of Nectin-4 expression within the tumor tissue, as has been previously demonstrated in research [19, 27, 30, 31]. Subsequent evaluations are necessary to ascertain whether there is an association with HPV, particularly in light of a recently published study that indicated a potentially heightened expression of Nectin-4 in HPV-positive tumors without evidence of a TP53 mutation [32].

For primary UC moderate and high expression of Nectin-4 were found in 51.5%. Nectin-4 expression decreased significantly during metastatic spread [17]. This was also observed in metastatic PC [19]. We observed a minimal decrease in Nectin-4 expression in recurrent or metastatic VSCC, but 28.8% of VSCC cases still exhibited moderate to strong Nectin-4 expression. Following the PFS results, VSCCs are in line with PCs and UCs with low Nectin-4 expression, indicating an unfavorable outcome [17]. A low expression or absence of Nectin-4 expression was also associated with a higher likelihood of resistance against EV [17]. Nevertheless, EV has been approved for mUC, regardless of Nectin-4 expression but the benefit of this ADC is only achieved in a small subset of patients [18]. The amplification of Nectin-4 is a stable genomic alteration that occurs during metastatic progression and is associated with high levels of Nectin-4 expression on the cell membrane. Objective responses to EV were seen in 96% of patients with Nectin-4 amplifications, compared with just 32% in the group with no amplification. Nectin-4 amplifications were found to reduce the risk of death by 92% in mUC [18]. In our study, none of the VSCC samples with high Nectin-4 expression showed clear or reliable amplification of Nectin-4. Different to UC vulvar cancers are mainly squamous cell carcinomas (VSCC). Clinical studies investigating possible objective response rates, PFS and OS in the therapy of EV for VSCC are needed to define subgroups that might benefit from the therapy of EV.

### Strength and limitations

This is the first and comparable large study investigating Nectin-4 expression in correlation with OS in women with VSCC. A potential disadvantage of the study lies in its retrospective design, which limits the accessibility of specific information, such as treatment modalities (e.g., surgery or radio- and chemoradiotherapy). Another potential drawback is the use of ngTMA instead of whole slide sections to determine the expression of Nectin-4, which can exhibit variability. To address this issue in a scientifically sound manner and following good research practice, we performed multiple core samples for each stain (at least two per person). For a subset of cases, whole slide staining and imaging were performed to validate homogeneity and comparison to ngTMA. This approach enabled us to thoroughly analyze a substantial number of VSCC cells. The use of ngTMA may explain different expression ratios compared to other studies. The present study also investigated the amplification of Nectin-4 in VSCC using FISH. We are the first study to evaluate the Nectin-4 expression and amplification of Nectin-4 in VSCC and the correlation of TILs. This facilitates a more profound comprehension of the nature of Nectin-4 in VSCC. Advanced tumors are most likely to be treated with systemic therapy, such as EV. The present collection of works is an accurate depiction of the clinical routine. The majority of vulvar cancers are diagnosed in the early stages. Due to the limited data available on Nectin-4 expression in VSCC and in order to represent a large collective, we analyzed all available samples. This should improve the understanding of Nectin-4 expression in VSCC. For other entities, such as breast cancer, neoadjuvant therapy approaches are now standard practice. In VSCC, these therapeutic approaches are not established due to a lack of data. Surgery can sometimes have a significant impact on women’s quality of life. Even with smaller tumors, the clitoris and/or urethra often have to be resected, sometimes with significant long-term consequences. This is why research on possible targets for systemic therapies is important for developing new therapeutic approaches, even for early stages of tumors.

## Conclusion

Therapy options for women with (advanced) VSCC are very limited. New therapy strategies are urgently needed. We therefore conducted this study to evaluate the expression of Nectin-4 in VSCC. We could show similar results of membranous Nectin-4 expression compared to PC, while cytoplasmic expression did not show significance or benefits. Nectin-4 H-score (membranous) does not significantly influence overall survival in addition to other prognostic factors. Nectin-4 was moderately and highly expressed in 32.4% of VSCC. In the subtype of p16+/p53- Nectin-4 expression was higher compared to other subtypes. Furthermore, the expression of Nectin-4 was observed in cases of recurrent or metastatic VSCC, a finding that contrasts with the expression patterns observed in PC. In mUC it has been shown that amplification of Nectin-4 correlates significantly better with response to EV. No amplification of Nectin-4 was detected in our study. However, the biological behavior of VSCC to mUC may be different as it involves squamous cell carcinomas rather than adenocarcinomas. Clinical studies are needed to investigate the potential clinical benefits of Nectin-4 targeted by EV.

## Supplementary Information

Below is the link to the electronic supplementary material.

ESM 1 (DOCX 19.6 MB)

## Data Availability

The data that support the findings of this study are available from the corresponding author upon reasonable request.
