# Supplementary material for: Nectin-4 expression in vulvar squamous cell carcinoma
Source: Virchows Arch. 2026 Mar 26;489(4):855–64. doi: 10.1007/s00428-026-04495-1 (PMC13714048; doi:10.1007/s00428-026-04495-1)
Supplement: Supplementary file 1 [file 428_2026_4495_MOESM1_ESM.docx]

|  |  | **All Patients**  **(N = 219)** |
| --- | --- | --- |
|  |  |  |
| Expression of CD 4+ | Mean (SD) | 216.6 (379.6) |
|  | Median (IQR) | 81.4 (21.8, 249.2) |
| Expression of CD 8+ | Mean (SD) | 478.3 (572.9) |
|  | Median (IQR) | 264.1 (85.0, 667.8) |
| Expression of CD 56+ | Mean (SD) | 299.9 (421.8) |
|  | Median (IQR) | 110.5 (45.7, 351.7) |
| Expression of CD 68+ | Mean (SD) | 454.8 (347.0) |
|  | Median (IQR) | 350.6 (209.4, 634.5) |
| Expression of FOXP3 | Mean (SD) | 62.1 (81.7) |
|  | Median (IQR) | 29.2 (13.6, 73.7) |

Table S1: Patient and tumour characteristics for tumor infiltrating lymphocytes (TIL’s) in patients with primary VSCC, showing mean and standard deviation (SD), median and interquartile range (IQR) or frequency and percentage

**Table S2:** Patient and tumor characteristics relative to Nectin 4 – H Score membranous (primary VSCC) for TIL’s

|  |  | **NECTIN 4- H Score (membranous)** | | | |
| --- | --- | --- | --- | --- | --- |
|  |  | **Negative (0 – 14)**  **(N = 98)** | **Weak (15 – 99)**  **(N = 50)** | **Moderate (100 – 199)**  **(N = 43)** | **Strong (200 – 300)**  **(N = 28)** |
| Expression of CD 4+ | Mean (SD) | 196.8 (326.8) | 225.2 (454.2) | 173.7 (194) | 331.5 (566.8) |
|  | Median (IQR) | 65 (14.8, 232.6) | 105.4 (24.2, 219.7) | 93.9 (14.8, 232.6) | 101.9 (24.2, 219.7) |
| Expression of CD 8+ | Mean (SD) | 493.2 (577.3) | 518.3 (644.7) | 365.9 (371.1) | 526.6 (675.9) |
|  | Median (IQR) | 325.8 (102.8, 650.9) | 317.8 (70.7, 678.6) | 217.8 (102.8, 650.9) | 142.1 (70.7, 678.6) |
| Expression of CD 56+ | Mean (SD) | 337.6 (494) | 245.4 (306.5) | 319.7 (415.1) | 240.2 (341.5) |
|  | Median (IQR) | 93.9 (34.9, 403.5) | 179.4 (76.9, 266) | 145.5 (34.9, 403.5) | 90.3 (76.9, 266) |
| Expression of CD 68+ | Mean (SD) | 540 (389.7) | 379.3 (290.8) | 423.4 (312.4) | 350.7 (278) |
|  | Median (IQR) | 426 (284.8, 744.9) | 306.5 (161.2, 506.8) | 339.8 (284.8, 744.9) | 285.6 (161.2, 506.8) |
| Expression of FOXP3 | Mean (SD) | 53.4 (66.4) | 61.2 (89.5) | 65.8 (81.9) | 87.4 (108.7) |
|  | Median (IQR) | 25.1 (9.6, 73.4) | 29.3 (19.4, 60.7) | 40 (9.6, 73.4) | 32.8 (19.4, 60.7) |

Figure S1: Further examples for well differentiated squamous cell cancer (keratinizing and non-keratinizing VSCC) as well as undifferentiated VSCC.

**a)**

**a)** Well differentiated keratinising squamous cell carcinoma, in which the NECTIN-4 staining shows both a high intensity membrane positivity and a low intensity cytoplasmatic positivity.

**b)**

**b)** In contrast, in this case the predominant staining pattern is cytoplasmatic (moderate to high intensity), even though a low intensity membrane positivity can also be identified.

**c)**

**c)** Poorly differentiated (G3) non keratinising squamous cell carcinoma composed of discohesive neoplastic cells surrounded by a fibromyxoid stroma. An example of how poorly differentiated tumours may lose NECTIN-4 expression. Notwithstanding, low intensity cytoplasmatic positivity is still retained in some cells.

**Figure S2a:** Recurrence-free survival rates relative to NECTIN 4- H Score (membraneous)

**Figure S2b:** Recurrence-free survival rates relative to NECTIN 4- H Score (cytoplasmatic)
